# Carotid vulnerable plaque in coronary heart disease: a machine learning-based diagnostic model integrating tongue parameters and blood metabolic biomarkers

**DOI:** 10.3389/fcvm.2026.1852366

**Published:** 2026-09-01

**Authors:** Xinang Xiao, Jieyun Li, Mi Zhou, Hui Gao, Qingsheng Wang, Yumo Xia, Jiekee Lim, Zhaoxia Xu

**Affiliations:** 1 School of Traditional Chinese Medicine, Shanghai University of Traditional Chinese Medicine (SHUTCM), Shanghai, China; 2 Key Laboratory of Health Identification and Assessment of Shanghai, Shanghai, China; 3 Guangde Traditional Chinese Medicine Clinic, Shanghai, China

**Keywords:** blood metabolic biomarkers, carotid plaque, coronary heart disease, diagnostic model, tongue manifestation parameters

## Abstract

**Purpose:**

This study aims to evaluate whether combining tongue manifestation parameters with blood metabolic biomarkers improves LASSO of carotid vulnerable plaques in patients with coronary heart disease (CHD), and to develop and compare the diagnostic performance of multiple machine-learning models.

**Methods:**

We retrospectively collected clinical data from 523 patients with CHD. Based on carotid ultrasound, patients were classified into vulnerable-plaque and non-vulnerable-plaque groups. We compared baseline characteristics, tongue manifestation parameters, and blood metabolic biomarkers between the two groups. The dataset was randomly split into training and validation sets at a 6:4 ratio. Factors associated with carotid vulnerable plaques in CHD were identified by intergroup comparison and LASSO regression, and four machine-learning algorithms were used to build predictive models.

**Results:**

Significant differences were found between the two groups in age, BMI,APOA1, FBG, AST, K^+^,Na^+^, BUN, PT, and APTT (*P* < 0.05). The R, G, and B values of the mid-tongue, tongue tip, right tongue, and the whole tongue also differed significantly between the vulnerable-plaque and non-vulnerable-plaque groups (*P* < 0.05). Tongue manifestation parameters correlated significantly with biomarkers including APOA1, FBG, PT, and APTT (*P* < 0.05). The LightGBM model has a high degree of discrimination in performance, with an AUC of 0.727 for the validation set.

**Conclusion:**

There is a weak correlation between tongue image parameters and some blood metabolic markers, suggesting that the two may reflect the pathophysiological state of the body from different dimensions. Tongue image parameters, as non-invasive and simple assessment indicators, provide a preliminary exploratory reference for the risk stratification of coronary heart disease. However, their independent predictive value and clinical practicability still need to be further verified by prospective large-sample studies.

## 1 Introduction

Coronary atherosclerosis is a principal pathological mechanism underlying coronary heart disease (CHD). Clinically, patients with CHD frequently exhibit concurrent carotid atherosclerosis (1). Carotid plaques develop through the persistent influence of multiple risk factors acting on the carotid vasculature (2). Carotid artery plaques can lead to lumen stenosis and induce hypoperfusion. Plaque rupture or detachment can cause distal arterial embolism, thereby inducing acute cardiovascular and cerebrovascular events (3). Among the many characteristics of carotid artery plaques, the vulnerability of the plaque (i.e., the tendency to rupture) is a better predictor of the risk of future cerebrovascular events than the simple degree of lumen stenosis (4). Therefore, early identification of whether there are vulnerable carotid plaques in patients with coronary heart disease is of great significance for the comprehensive prevention and control of cardiovascular and cerebrovascular events.

Carotid ultrasound is currently the main method for diagnosing carotid artery plaques and can provide valuable morphological information. However, this method has the following limitations: First, the ultrasound assessment results are greatly influenced by the operator's experience, and the consistency of diagnosis needs to be improved; Second, ultrasound examination only provides structural information and is difficult to reflect the systemic metabolic disorders and inflammatory states behind the vulnerability of plaques. Thirdly, in primary medical institutions, the equipment cost and technical threshold of high-frequency carotid ultrasound have restricted its promotion as a large-scale screening tool. Therefore, it is urgently necessary to explore a simple, non-invasive, low-cost auxiliary assessment method that can comprehensively reflect the pathophysiological state of the whole body for the early identification and risk stratification of carotid vulnerable plaques in patients with coronary heart disease.

Tongue diagnosis, a distinctive and noninvasive method in Traditional Chinese Medicine (TCM), infers internal physiological states from tongue color and texture and thus holds diagnostic promise. Advances in digital image processing now allow objective quantification of tongue features, adding a measurable dimension to disease assessment (5, 6). Studies show that these objective tongue parameters differ across TCM syndrome types in CHD, indicating potential for quantitative TCM pattern diagnosis (7). Tongue characteristics also correlate with coronary lesion location, and shifts in tongue appearance can partially reflect vascular pathology severity (8). However, most of the above-mentioned studies are limited to descriptive analysis at a single time point and have not systematically explored the association between tongue image parameters and the specific clinical outcome of carotid plaque vulnerability.

Blood metabolic biomarkers closely relate to coronary and carotid atherosclerosis, since carotid plaque formation is driven by factors such as dyslipidemia, hyperglycemia, and inflammation (9–11). In recent years, the integrated analysis of multi-dimensional biomarkers has made certain progress in the risk assessment of atherosclerosis. For example, combined metabolic indicators have been used to predict new-onset carotid plaques (12), AI-ECG age has been found to be independently associated with carotid plaque volume and progression (13), and the association between cerebrovascular risk factors and metabolic disorders has also received attention (14). Machine learning has advanced medical research, yet only a few studies have built predictive models for carotid plaques (15, 16). However, most previous studies have included modern medical indicators and have not yet incorporated objective parameters of tongue appearance in traditional Chinese medicine and blood metabolism markers. Although tongue images and blood metabolic markers each have potential, their combined application in evaluating carotid vulnerable plaques in patients with coronary heart disease remains rarely explored.

**Figure 1:**
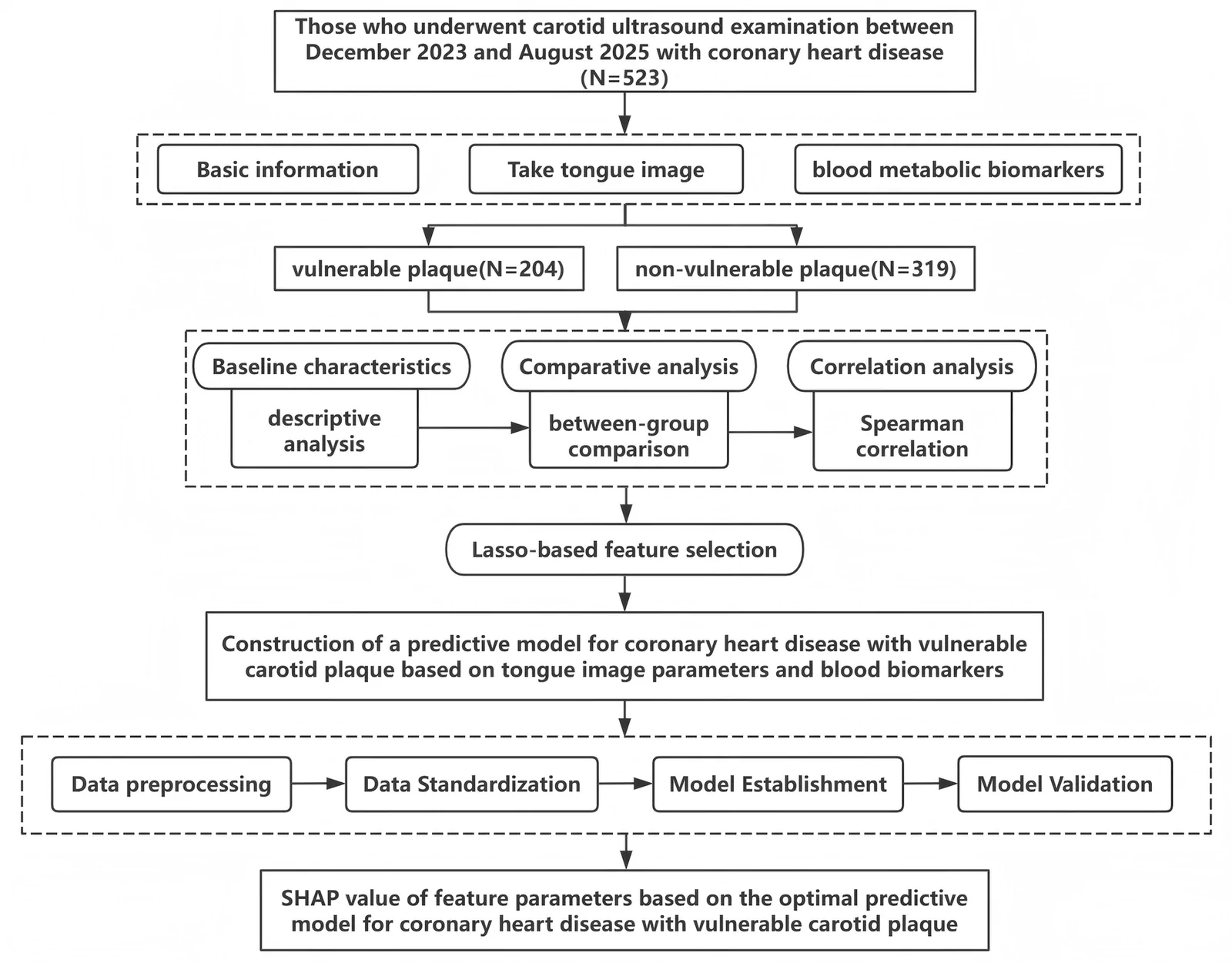
Flow chart.

Based on the above background, the existing research has the following gaps: There is a lack of predictive tools for carotid vulnerable plaques in patients with coronary heart disease, a specific high-risk group. Previous studies have mostly focused on the presence of plaques rather than plaque vulnerability. The existing LASSO models mainly rely on modern medical indicators and have not yet incorporated the objective parameters of traditional Chinese medicine tongue diagnosis into the multimodal LASSO framework. The intrinsic relationship between tongue image parameters and blood metabolic markers and their combined predictive value remain unclear. Therefore, this study intends to compare the differences in tongue image parameters and metabolic indicators between the vulnerable plaque group and the non-vulnerable plaque group in patients with coronary heart disease. Analyze the correlation between tongue image parameters and metabolic indicators; Construct and compare multiple machine learning models to evaluate the predictive efficacy of their combined application for vulnerable carotid artery plaques. This study aims to explore a non-invasive assessment tool that integrates objective parameters of traditional Chinese tongue diagnosis with modern biomarkers, providing a preliminary exploratory basis for cardiovascular risk stratification in patients with coronary heart disease. The detailed flow chart of this study is shown in Figure 1.

## 2 Material and methods

### 2.1 Study population

We retrospectively collected clinical data from 523 patients with CHD who underwent carotid ultrasound at Shuguang Hospital, Yueyang Hospital of Integrated Traditional Chinese and Western Medicine, and Jiading District Central Hospital in Shanghai between December 2023 and August 2025. For all patients we obtained complete demographic information, tongue manifestation parameters, and biochemical indicators. Written informed consent was obtained from each participant, and personal data were kept strictly confidential and used only for this research. The study was approved by the Ethics Committee of Shanghai University of Traditional Chinese Medicine (Approval No.: 2023-3-10-08-07).

### 2.2 Inclusion and exclusion criteria

Inclusion criteria: (1) Meets Western medical diagnostic criteria for CHD and the diagnostic criteria for carotid plaque; (2) age 35–80 years, any sex; (3) complete carotid ultrasound report, tongue images, and clinical laboratory test results available; (4) informed consent obtained and signed.

Exclusion criteria: (1) impaired consciousness, cognitive dysfunction, communication barriers, or hearing disorders; (2) severe organic disease affecting the brain, lungs, kidneys, liver, or other vital organs; (3) pregnancy or diagnosed mental disorder; (4) severely incomplete clinical data.

The Western medical diagnosis of CHD in this study followed the 2018 “Guideline for the Diagnosis and Treatment of Stable Coronary Artery Disease” issued by the Chinese Society of Cardiology, Chinese Medical Association (17), and included chronic stable exertional angina, ischemic cardiomyopathy, and the stable phase after treatment for acute coronary syndrome.

In this study, the diagnosis of carotid artery plaques in coronary heart disease referred to the “Expert Consensus on Several Issues of Head and Neck Vascular Ultrasound (Carotid Artery Part)” (18), and the plaque assessment was independently conducted by two ultrasound physicians. When two ultrasound physicians were conducting carotid artery plaque assessment, they were completely unaware of the patient's clinical data (including tongue image parameters and laboratory test results), and only judged the nature of the plaque based on ultrasound images. Carotid artery plaques are classified into vulnerable plaques and non-vulnerable plaques. Their ultrasound manifestations and plaque types are as follows. Vulnerable plaques: The plaque shape is irregular, the surface fiber cap is discontinuous or broken, the texture is relatively soft, and low echo is observed. Heterogeneous plaque: The surface of the plaque is irregular and may be accompanied by rupture or ulceration of the fibrous cap. The echo within the plaque presents a mixed echo, or low echo and strong echo occur simultaneously. When a patient has multiple carotid artery plaques, as long as any one of them is determined to be a vulnerable plaque, the patient is classified into the vulnerable plaque group. Non-vulnerable plaque: Stable plaque: The plaque has a regular shape, with a continuous and smooth fibrous cap on the surface, relatively hard texture, and strong echo or isoecho is observed.

### 2.3 Data acquisition and extraction

#### 2.3.1 Clinical index collection

Data were collected from 523 patients undergoing coronary angiography. Basic demographics and clinical variables—age, gender, BMI, past medical history, and blood metabolic markers (e.g., TC, TG, HDL-C, LDL-C, APOA1, FBG, HBA1C, AST, ALT, K^+^, Na^+^, Cl−, BUN, Cr, UA, D-dimer, PT, APTT, PTA, INR, FIB)—were recorded, along with carotid ultrasound results. Carotid ultrasonography was performed by professional radiologists following standard operating procedures to ensure accuracy, and the findings were entered into a unified clinical investigation form to support subsequent data analysis.

#### 2.3.2 Tongue image parameters collection

Tongue images were acquired with a Canon PowerShot SX720 HS digital camera and a color card. Participants abstained from drinking for 30 min and from eating for 1 h. After resting for at least 5 min, they sat or lay down, relaxed their facial muscles, opened their mouths, and extended their tongues for 3 s while images were taken (19). Four experienced traditional Chinese medicine practitioners (JY-L, LX-H, XA-X, and R-G) collected tongue-diagnostic parameters within 6 h before coronary angiography.

#### 2.3.3 Tongue image parameters extraction

**Figure 2:**
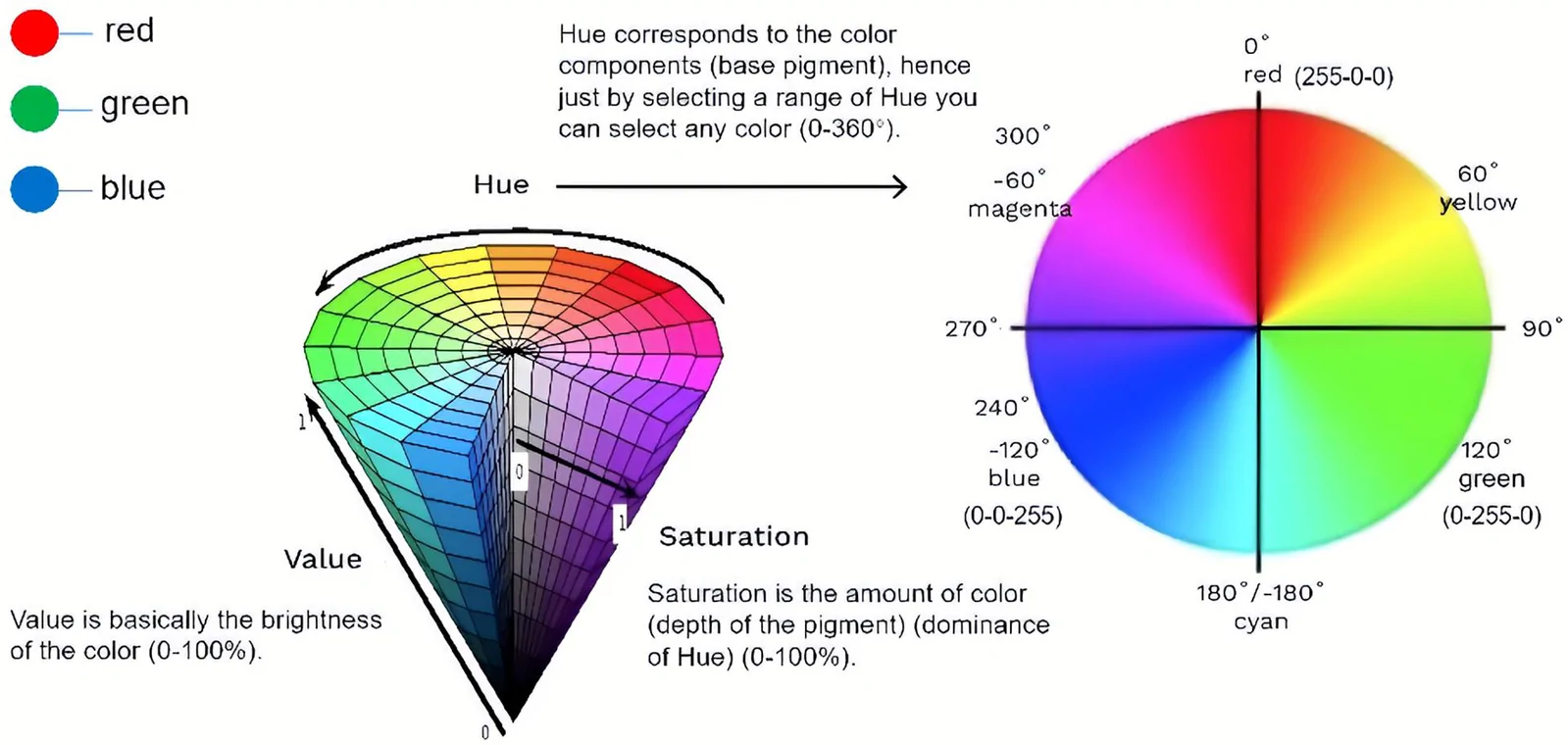
RGB and HSV color model.

**Figure 3:**
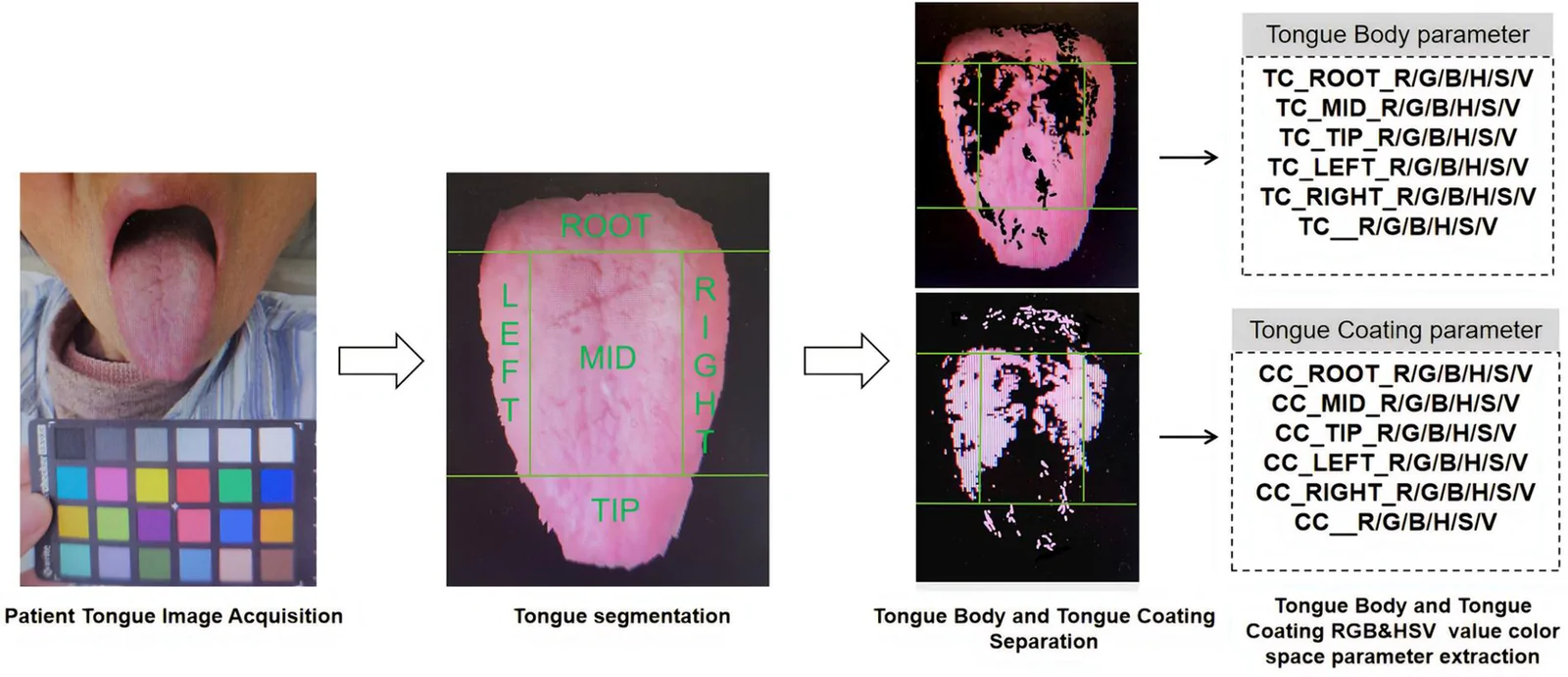
Schematic diagram of tongue image parameter extraction.

The tongue tissue and tongue coating were separated and segmented using SMX System 2.0 tongue image analysis software (Registration No. 2008SR12316). Color parameters for both tissue and coating were quantified using the RGB and HSV models. As shown in Figure 2, the RGB model is an additive color model that encodes colors by varying the intensities of three primaries—red, green, and blue—each expressed as an integer from 0 to 255, where 0 indicates absence and 255 indicates maximum intensity (20). By contrast, the HSV model is organized around human visual perception; the H component (hue) denotes basic color categories such as red, green, and blue, S (saturation) denotes color purity, and V (value) denotes lightness (21). The tongue's surface is complex, being covered with papillae and taste buds, and different regions can display distinct features under various diseases or pathological processes. As illustrated in Figure 3, to facilitate systematic observation and analysis, the tongue was partitioned into specific regions.

### 2.4 Machine learning model development

We evaluated four machine-learning algorithms—logistic regression, LightGBM, XGBoost, and support vector machine (SVM)—to build a predictive model for coronary heart disease with carotid vulnerable plaque. These algorithms were chosen for their complementary strengths in handling clinical and high-dimensional data and for their validated performance in prior medical classification tasks (22–24).

**Logistic Regression:** A model estimating binary outcomes using logistic function, selected for its stability and interpretability.
**LightGBM:** A gradient boosting framework optimized for histograms, ideal for mixed clinical-imaging data and imputation of missing values.
**XGBoost:** A scalable boosting system with regularization for balancing model complexity.
**SVM:** A kernel-based classifier using radial basis functions to capture nonlinear feature interactions.

In this study, During each model construction process, the dataset is randomly sampled in a 6:4 ratio and divided into a training set (*n* = 313) and a validation set (*n* = 210). The training set is used to build the model, while the validation set is used to verify the model's performance. To reduce the influence of dimensions, all continuous variables were Z-score standardized before modeling. LASSO regression is strictly implemented within the training set (*λ* values are selected through 10-fold cross-validation), and then the selected features are applied to independent validation sets. Based on the feature variables screened out by LASSO, ensure that the distribution ratios of each group of plaque types in the training set and validation set are consistent with the original dataset. During the model construction process, ten-fold cross-validation is used to internally validate the training set. The data is calculated based on the ten-fold cross-validation results on the training set, with SD as the standard deviation. The hyperparameters of each model are optimized through grid search.

### 2.5 Statistical analysis

Statistical analyses employed chi-square tests, Welch's *t*-tests, and Fisher's exact tests, performed in R (v4.2) and IBM SPSS (25.0). All clinical indicators and tongue parameters were Z-score normalized. Continuous data are reported as mean ± standard deviation (SD) or median (interquartile range, IQR). Group differences were evaluated with chi-square or t-tests, using two-tailed tests and a significance threshold of *p* < 0.05. Associations were assessed with Spearman's correlation, and correlation heatmaps were produced using the online plotting tool (https://www.chiplot.com).

## 3 Results

### 3.1 Basic information

A total of 523 patients with CHD were included: 204 (39%) in the vulnerable plaque group and 319 (61%) in the non-vulnerable plaque group. Significant differences between the groups were found for age, BMI, FBG, AST, Na^+^, PT, and APTT (*P* < 0.05). Patients in the vulnerable plaque group were younger and had higher BMI than those in the non-vulnerable plaque group. With respect to blood metabolic biomarkers, the vulnerable plaque group showed higher FBG, AST, Na^+^, PT, and APTT and lower APOA1, K^+^, and BUN compared with the non-vulnerable plaque group. No statistically significant differences were observed for sex, smoking history, alcohol consumption history, past medical history, or other blood metabolic biomarkers (*P* > 0.05). Details are shown in Table 1.

**Table 1:** Basic characteristic information of patients.

Factor	Vulnerable plaque (*n* = 204)	Non-vulnerable plaque (*n* = 319)	Statistic	*P*
Age (year)	70.00 (64.00, 75.00)	72.00 (67.00, 77.00)	2.866	0.004**
Sex (%)			0.380	0.538
Male	106 (51.96)	174 (54.71)		
Female	98 (48.04)	144 (45.29)		
BMI (kg/m^2^)	24.77 (22.49, 26.78)	23.12 (22.04, 25.66)	−2.916	0.004**
SH *n* (%)	89 (43.62)	158 (49.53)	1.739	0.187
LDH *n* (%)	89 (43.62)	153 (47.96)	0.941	0.332
PMH *n* (%)				
Hypertension	129 (63.23)	165 (67.90)	1.072	0.300
Diabetes	72 (35.29)	78 (32.09)	0.508	0.476
Hyperlipidemia	21 (10.44)	28 (11.66)	0.165	0.685
Hyperuricemia	8 (3.92)	9 (3.70)	0.014	0.905
Cerebral infarction	26 (12.74)	31 (12.75)	0.000	0.997
Arrhythmia	18 (8.82)	19 (7.81)	0.147	0.701
TC (mmol/L)	3.81 (3.30, 4.55)	3.70 (3.10, 4.60)	−1.851	0.064
TG (mmol/L)	1.30 (0.95, 1.91)	1.31 (0.95, 1.76)	−0.740	0.460
HDL-C (mmol/L)	1.04 (0.89, 1.24)	1.10 (0.93, 1.27)	1.719	0.086
LDL-C (mmol/L)	2.25 (1.80, 2.98)	2.20 (1.76, 2.93)	−0.638	0.523
APOA1 (g/L)	1.06 (0.92, 1.24)	1.11 (0.99, 1.25)	2.009	0.045*
FBG (mmol/L)	5.70 (4.98, 7.20)	5.40 (4.72, 6.44)	−2.898	0.004**
HBA1C (mmol/L)	6.10 (5.60, 6.90)	6.10 (5.70, 6.90)	0.485	0.628
AST (U/L)	24.00 (19.00, 30.00)	21.00 (18.00, 28.00)	−2.447	0.014*
ALT (U/L)	21.23 (14.20, 28.28)	19.36 (14.00, 31.00)	−0.298	0.766
K^+^ (mmol/L)	3.92 (3.68, 4.14)	3.97 (3.71, 4.27)	2.004	0.045*
Na^+^ (mmol/L)	140.0 (138.0, 142.0)	139.2 (137.0, 141.0)	−2.586	0.010*
Cl− (mmol/L)	105.2 (103.5, 108.0)	106.0 (103.5, 108.0)	0.348	0.728
BUN (mmol/L)	6.10 (5.10, 7.59)	6.50 (5.21, 8.45)	2.007	0.045*
Cr (mmol/L)	72.50 (60.60, 84.70)	72.20 (61.30, 86.40)	0.425	0.671
UA (mmol/L)	336.2 (257.3, 400.1)	330.5 (264.8, 395.0)	−0.487	0.626
D-dimer (mg/L)	0.36 (0.21, 0.55)	0.32 (0.21, 0.56)	−0.782	0.434
PT (s)	11.70 (11.20, 12.60)	11.40 (10.80, 12.40)	−2.589	0.010*
APTT (s)	27.10 (25.50, 29.60)	26.40 (25.20, 28.20)	−2.497	0.013*
PTA (%)	97.70 (88.00, 108.8)	100.0[91.90, 110.5]	0.674	0.501
INR	1.02 (0.95, 1.07)	1.01 (0.94, 1.07)	−0.192	0.848
FIB (g/L)	2.84 (2.45, 3.39)	2.98 (2.53,3.47)	1.790	0.073

* *P* < 0.05.

** *P* < 0.01.

### 3.2 Tongue color parameter features of classified patients

Regarding tongue color parameters, the vulnerable plaque group exhibited an increasing trend in the R, G, and B values across various regions of the tongue, including the middle, tip, right side, and the overall tongue area (*P* < 0.01), indicating a brighter tongue color. Additionally, in the HSV color space, both the S and V values significantly increased in multiple tongue regions (*P* < 0.05), reflecting enhanced saturation and brightness of the tongue color. Detailed results are presented in Table 2.

**Table 2:** Tongue color differences in patients with vulnerable vs. stable carotid plaques.

Regions	TC	Vulnerable plaque (*n* = 204)	Non-vulnerable plaque (*n* = 319)	*P*
Root	R	128.1 (109.8, 146.6)	131.8 (117.4, 152.2)	0.021*
	G	86.21 (66.01, 102.8)	90.57 (77.33, 109.1)	0.005**
	B	88.16 (68.32, 106.2)	90.98 (74.75, 108.6)	0.066
	H	340.2 (2.53, 355.7)	6.00 (2.01, 356.2)	0.248
	S	0.33 (0.26, 0.40)	0.30 (0.24, 0.38)	0.037*
	V	0.50 (0.43, 0.57)	0.51 (0.46, 0.59)	0.029*
Middle	R	185.2 (164.1, 206.8)	174.5 (161.3, 186.7)	<0.001**
	G	123.4 (108.6, 141.3)	119.3 (104.5, 132.9)	0.006**
	B	129.1 (113.6, 147.0)	120.9 (106.4, 134.4)	<0.001**
	H	351.3 (323.1, 356.5)	354.4 (7.12, 357.6)	0.022*
	S	0.32 (0.26, 0.38)	0.31 (0.26, 0.37)	0.426
	V	185.2 (164.1, 206.8)	174.5 (161.3, 186.7)	<0.001**
Tip	R	179.3 (161.1, 197.3)	164.1 (147.6, 181.0)	<0.001**
	G	103.4 (89.86, 116.1)	95.63 (81.84, 109.2)	<0.001**
	B	110.2 (95.94, 122.7)	98.86 (86.37, 113.0)	<0.001**
	H	352.0 (344.0, 355.7)	354.7 (350.5, 357.2)	<0.001**
	S	0.42 (0.35, 0.47)	0.41 (0.37, 0.46)	0.874
	V	179.0 (161.1, 197.3)	164.1 (147.6, 181.0)	<0.001**
Left	R	161.7 (141.1, 186.9)	158.4 (143.1, 175.6)	0.223
	G	100.7 (81.71, 121.3)	100.9 (84.59, 114.8)	0.889
	B	105.4 (86.54, 127.1)	101.1 (86.08, 118.1)	0.152
	H	352.1 (338.0, 355.9)	354.3 (4.79, 357.4)	0.042*
	S	0.37 (0.31, 0.44)	0.37 (0.32, 0.42)	0.518
	V	161.7 (140.9, 186.9)	158.4 (143.1, 175.6)	0.286
Right	R	175.0 (154.2, 198.6)	170.5 (148.8, 188.0)	0.005**
	G	116.0 (98.96, 131.8)	114.6 (94.26, 131.08)	0.351
	B	119.6 (101.5, 139.6)	116.5 (96.95, 133.3)	0.035*
	H	350.9 (8.13, 356.1)	355.0 (6.63, 357.8)	<0.001**
	S	0.33 (0.28, 0.40)	0.32 (0.28, 0.38)	0.402
	V	175.0 (153.6, 198.6)	170.5 (148.8, 188.0)	0.008**
Whole	R	170.0 (151.1, 182.7)	164.4 (151.6, 176.4)	0.011*
	G	108.1 (93.38, 121.4)	108.1 (95.22, 119.8)	0.802
	B	113.1 (97.54, 124.9)	109.1 (97.20, 120.2)	0.052
	H	352.9 (334.2, 356.7)	356.2 (12.81, 358.3)	<0.001**
	S	0.35 (0.29, 0.40)	0.34 (0.29, 0.39)	0.202
	V	169.8 (150.4, 182.7)	164.4 (151.6, 176.4)	0.018*

TC, represents the tongue color; ROOT, LEFT, RIGHT, MID, TIP, represent different parts of the tongue respectively.

No adjustment was made for multiple testing, and all comparisons should be considered exploratory analyses.

* *P* < 0.05.

** *P* < 0.01.

### 3.3 Tongue coating color parameter features of classified patients

Regarding tongue-coating color parameters, the vulnerable-plaque group showed significantly higher R values in the root, mid-tongue, tip, left, right, and whole-tongue regions than the control group (*P* < 0.001). G and B values were also significantly higher in most regions (*P* < 0.05), indicating a redder coating. Detailed results are shown in Table 3.

**Table 3:** Tongue coating color differences in patients with vulnerable vs. stable carotid plaques.

Regions	CC	Vulnerable plaque (*n* = 204)	Non-vulnerable plaque (*n* = 319)	*P*
Root	R	144.4 (125.3, 170.9)	136.6 (119.7, 154.3)	<0.001**
	G	102.5 (87.87, 128.6)	99.40 (83.42, 118.3)	0.058
	B	104.0 (81.73, 133.0)	96.54 (79.41, 116.7)	0.002**
	H	320.0 (11.68, 345.6)	21.06 (11.34, 347.5)	0.647
	S	0.31 (0.22, 0.38)	0.30 (0.24, 0.38)	0.610
	V	143.8 (125.2, 170.6)	136.6 (120.2, 154.3)	<0.001**
Middle	R	190.8 (168.7, 216.1)	181.6 (166.5, 191.9)	<0.001**
	G	137.1 (118.3, 155.7)	128.4 (114.6, 144.0)	<0.001**
	B	141.3 (123.0, 164.1)	133.0 (116.6, 148.0)	<0.001**
	H	340.5 (19.14, 347.7)	345.6 (17.45, 350.4)	0.032*
	S	0.29 (0.23, 0.35)	0.30 (0.24, 0.35)	0.325
	V	190.8 (168.7, 216.4)	181.6 (166.6, 191.9)	<0.001**
Tip	R	186.8 (163.4, 210.6)	169.4 (149.0, 187.5)	<0.001**
	G	118.2 (95.39, 136.4)	105.5 (88.47, 122.3)	<0.001**
	B	128.2 (102.3, 148.4)	113.4 (95.28, 130.3)	<0.001**
	H	345.0 (315.2, 349.4)	347.2 (15.25, 351.0)	0.053
	S	0.35 (0.29, 0.41)	0.36 (0.29, 0.42)	0.660
	V	186.8 (163.4, 210.6)	169.4 (149.0, 187.5)	<0.001**
Left	R	176.6 (146.1, 205.8)	162.3 (138.4, 179.5)	<0.001**
	G	119.7 (95.63, 143.8)	109.9 (87.63, 128.7)	<0.001**
	B	128.8 (99.76, 155.8)	112.4 (85.23, 135.1)	<0.001**
	H	341.1 (15.19, 348.2)	343.0 (10.55, 350.3)	0.691
	S	0.31 (0.24, 0.38)	0.32 (0.26, 0.38)	0.236
	V	172.3 (145.7, 205.8)	162.3 (138.4, 179.5)	<0.001**
Right	R	188.4 (162.2, 217.1)	174.4 (153.0, 192.8)	<0.001**
	G	129.7 (109.7, 156.6)	125.6 (102.3, 141.2)	<0.001**
	B	140.6 (113.3, 170.4)	128.3 (104.5, 147.1)	<0.001**
	H	340.5 (14.95, 347.2)	344.7 (13.13, 350.4)	0.032*
	S	0.28 (0.22, 0.35)	0.30 (0.23, 0.34)	0.778
	V	188.4 (162.1, 217.1)	174.4 (153.0, 192.8)	<0.001**
Whole	R	180.8 (159.0, 207.1)	169.8 (153.9, 181.9)	<0.001**
	G	128.5 (109.3, 144.2)	121.1 (106.6, 133.5)	<0.001**
	B	132.9 (113.8, 152.6)	122.6 (105.8, 138.3)	<0.001**
	H	341.6 (24.50, 348.4)	345.1 (15.65, 350.9)	0.228
	S	0.30 (0.23, 0.36)	0.31 (0.26, 0.35)	0.396
	V	180.3 (158.0, 208.7)	169.8 (154.1, 181.9)	<0.001**

CC, represents the tongue coating color;ROOT, LEFT, RIGHT, MID, TIP, represent different parts of the tongue respectively.

No adjustment was made for multiple testing, and all comparisons should be considered exploratory analyses.

* *P* < 0.05.

** *P* < 0.01.

### 3.4 Spearman correlation analysis

**Figure 4:**
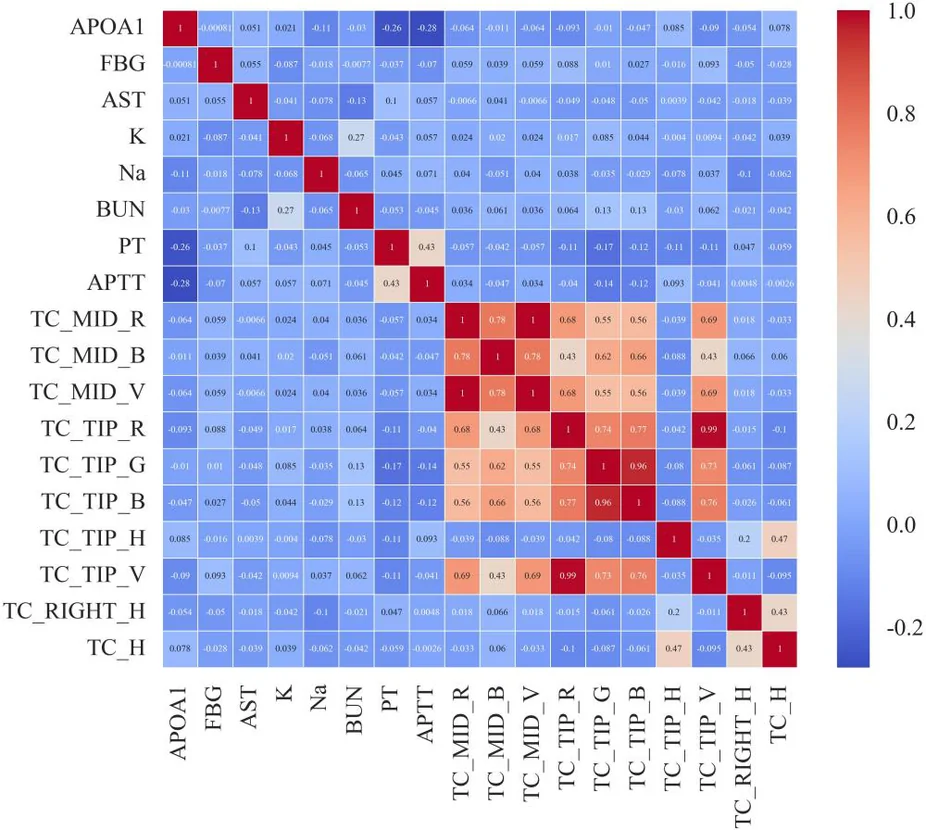
Correlation of tongue color parameters and blood metabolic biomarkers.

Figure 4 presents the correlation heatmap of tongue color parameters and blood metabolic markers. Spearman correlation analysis showed that there was a statistically weak correlation between tongue color parameters and multiple blood metabolic markers, suggesting that the linear association strength between tongue image parameters and metabolic indicators was relatively weak, and the two might reflect relatively independent biological information dimensions. TC_MID_R was negatively correlated with APOA1 (r = −0.064) and positively correlated with FBG (r = 0.059). BTC_MID_V correlated with BUN (r = 0.13) and with PT (r = −0.17). TC_MID_R and VTC_RIGHT showed positive correlations with K^+^ (r = 0.024 and r = 0.085, respectively). APTT correlated positively with BTC_MID_V (r = 0.12) and with VTC_RIGHT (r = 0.093). Strong collinearity was also observed among several tongue manifestation parameters, notably TC_MID_R, BTC_MID_V, and VTC_RIGHT.

**Figure 5:**
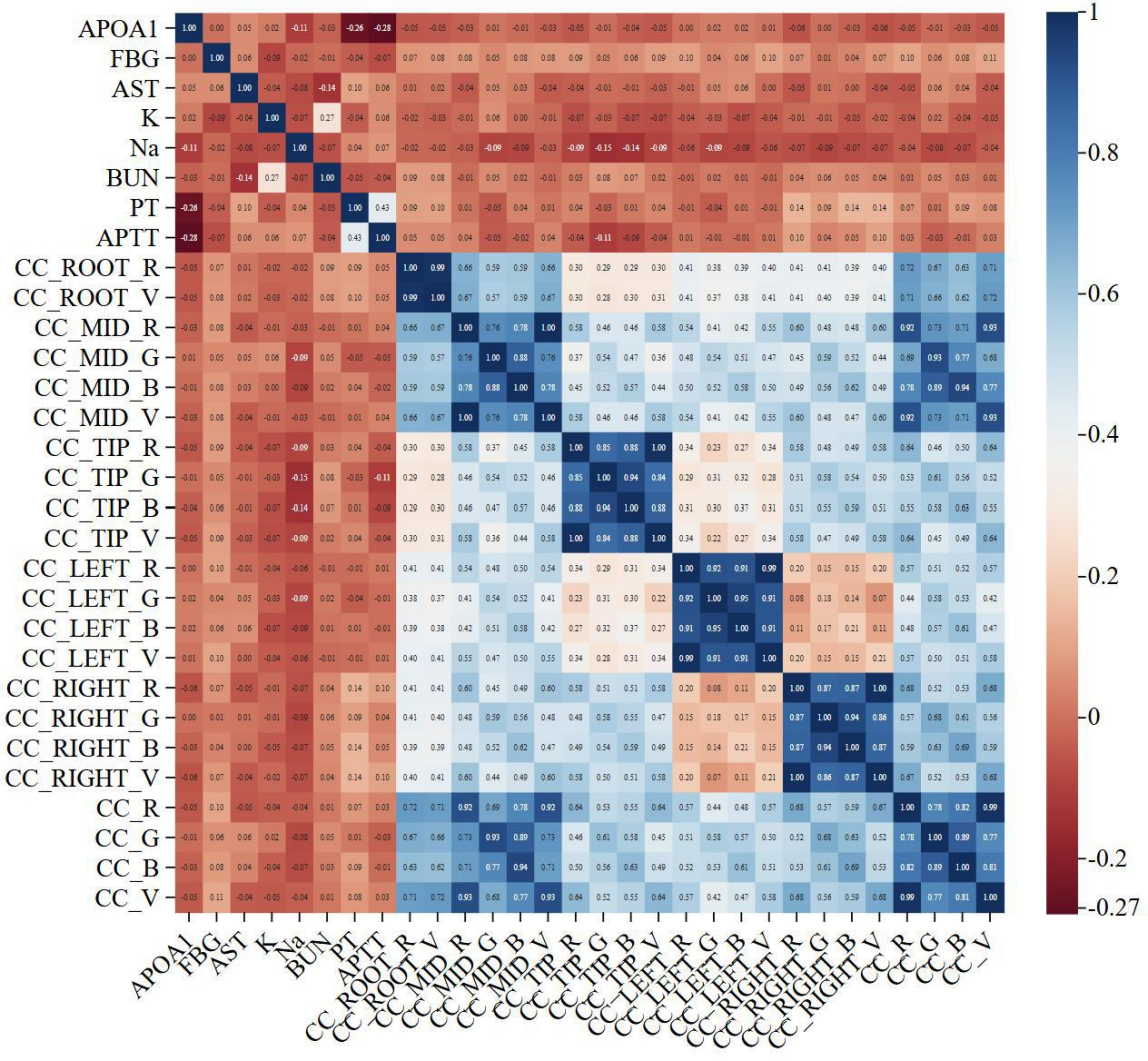
Correlation of tongue coating color parameters and blood metabolic biomarkers.

Figure 5 presents the correlation heatmap between tongue-coating color parameters and blood metabolic markers. The correlation between tongue coating color parameters and blood metabolic markers also shows similar characteristics, with a weak correlation level. Although these weak correlations are statistically significant, their effect sizes are small and do not support the inference of strong causal associations.CC_ROOT_V had a weak positive correlation with BUN (r = 0.10) and with FBG (r = 0.06). CC_MID_B also exhibited a weak positive correlation with BUN (r = 0.08). CC_ROOT_R showed a weak positive correlation with K^+^ (r = 0.05). CC_TIP_G had a weak negative correlation with AST (r = −0.05), and CC_RIGHT_G had a weak negative correlation with APTT (r = −0.05).

### 3.5 Model development and validation

**Figure 6:**
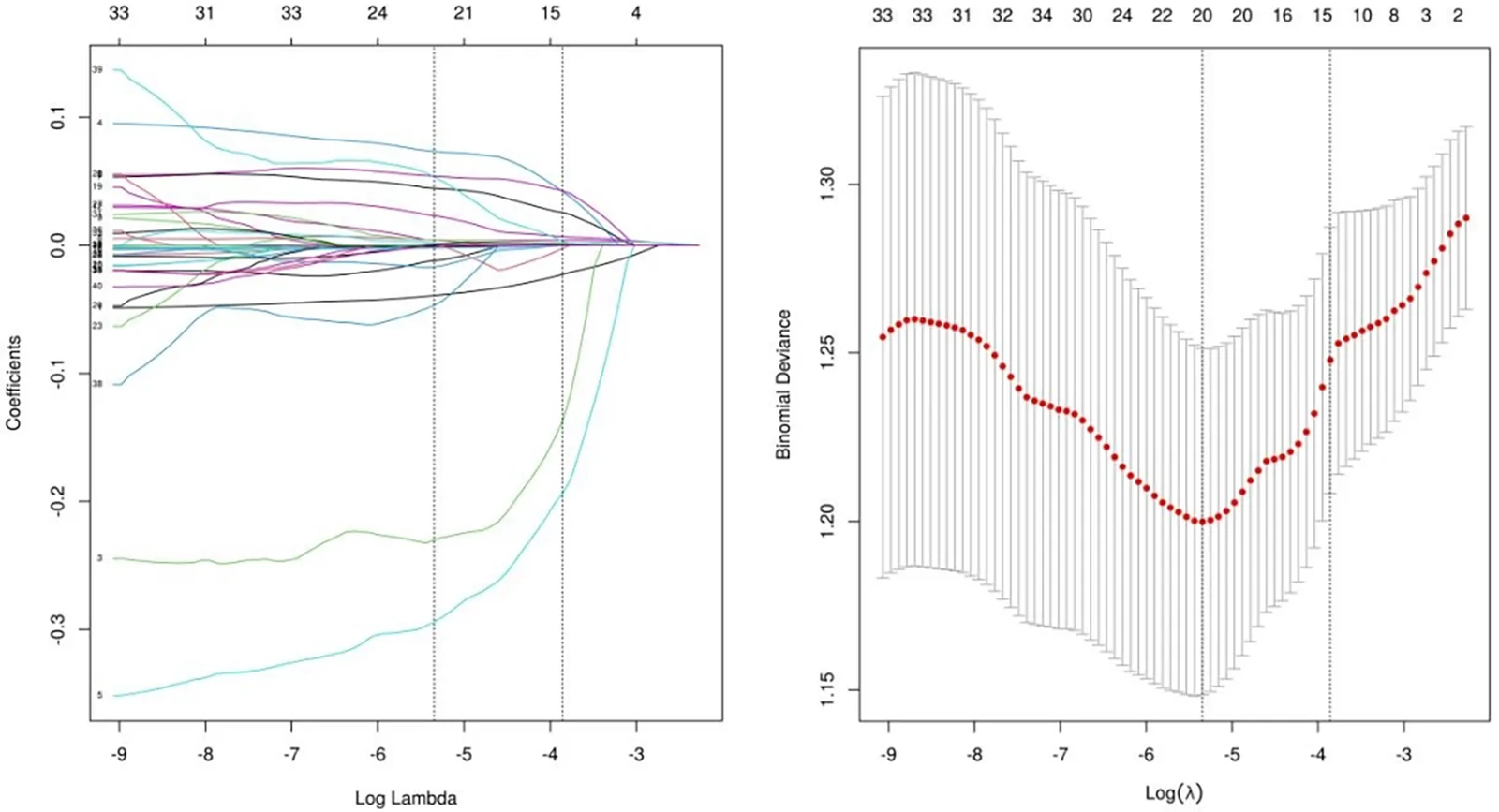
LASSO regression coefficient path plot and LASSO regression cross-validation plot.

We identified the most informative predictors of carotid vulnerable plaque using LASSO regression (Figure 6). A penalization parameter (*λ*) was selected by cross-validation to minimize binomial deviance, and features with nonzero coefficients were retained for model construction. Fourteen features were selected: age, APOA1, FBG, K, Na, PT, TC_TIP_R, TC_TIP_H, TC_H, CC_MID_G, CC_TIP_B, CC_LEFT_B, CC_RIGHT_B, and CC_B. These features will be used to build machine-learning models in the next stage, providing a streamlined and interpretable basis for optimizing classification performance.

To evaluate whether combining tongue manifestation parameters with blood metabolic biomarkers improves discrimination of carotid plaque stability in CHD patients, diagnostic models were developed from the 14 features with non-zero coefficients selected by LASSO regression. The dataset was randomly split into a training set (*n* = 313) and a validation set (*n* = 210) at a 6:4 ratio. Four algorithms—Logistic Regression, XGBoost, LightGBM, and SVM—were used to build the models, and performance was internally validated using ten-fold cross-validation.

In the training set, all four models demonstrated excellent discriminatory abilities. Among them, the AUC values of the XGBoost and LightGBM models both reached 1.000, and their accuracy, sensitivity, specificity and F1 score were all 1.000, suggesting that they have perfect fitting ability in the training set. The SVM model also performed well, with an AUC of 0.891 (SD = 0.009) and an accuracy of 0.854 (SD = 0.013). The AUC of the Logistic regression model was 0.742 (SD = 0.033), and the accuracy was 0.719 (SD = 0.029). Details are presented in Table 4.

**Table 4:** Results on the classification of machine learning model on training set.

Model	AUC (SD)	Accuracy (SD)	Sensitivity (SD)	Specificity (SD)	F1Score (SD)	Kappa (SD)
Logistic	0.742 (0.010)	0.719 (0.029)	0.580 (0.072)	0.790 (0.076)	0.583 (0.022)	0.373 (0.036)
XGBoost	1.000 (0.000)	1.000 (0.000)	1.000 (0.000)	1.000 (0.000)	1.000 (0.000)	1.000 (0.000)
LightGBM	1.000 (0.000)	1.000 (0.000)	1.000 (0.000)	1.000 (0.000)	1.000 (0.000)	1.000 (0.000)
SVM	0.891 (0.009)	0.854 (0.013)	0.829 (0.036)	0.866 (0.036)	0.795 (0.011)	0.681 (0.022)

**Figure 7:**
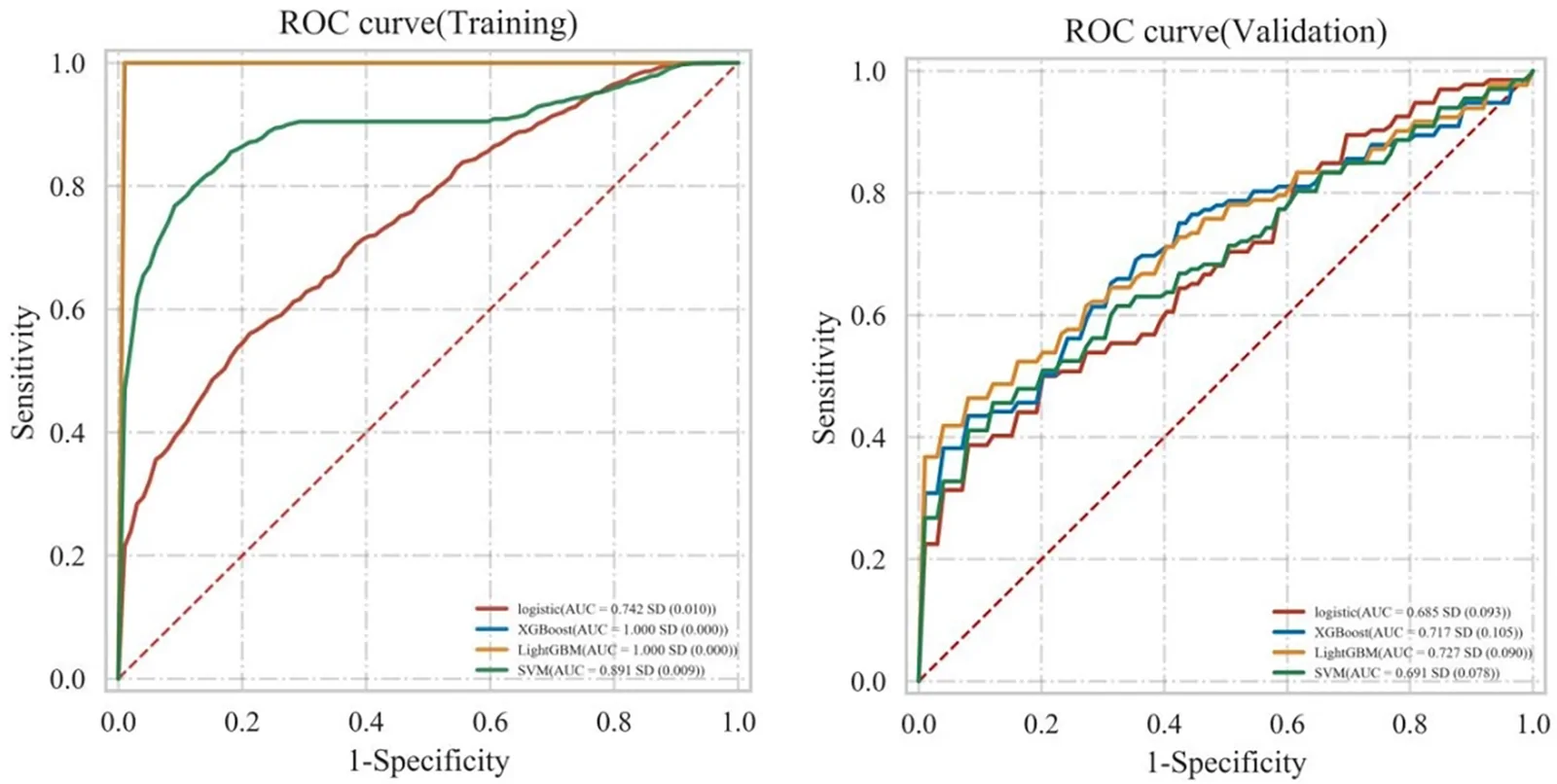
Receiver operating characteristic (ROC) curves of the four machine learning models in the training and validation cohorts.

**Figure 8:**
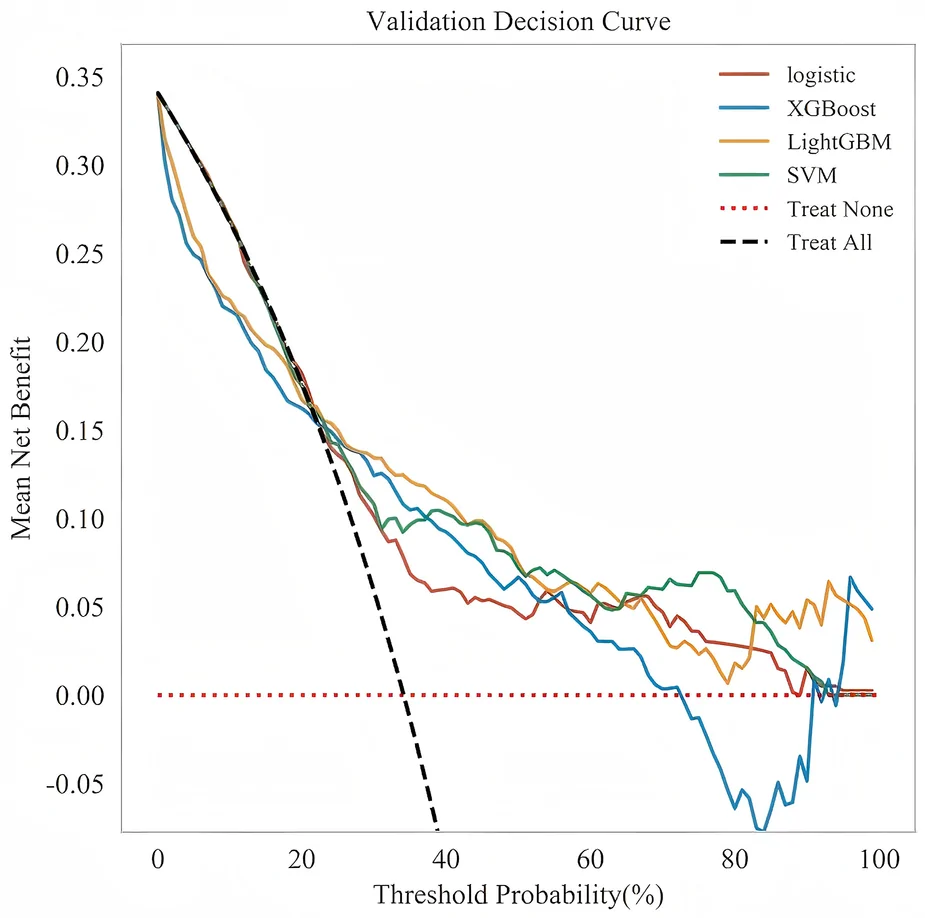
The DCA curves of vulnerable carotid plaque were verified by different models.

In the validation set, the generalization ability of the models shows certain differences. The XGBoost model still maintains a high diagnostic efficiency, with an AUC of 0.717 (SD = 0.105) and an accuracy of 0.738 (SD = 0.034). The AUC of the LightGBM model was 0.727 (SD = 0.041), and the accuracy was 0.751 (SD = 0.041). The AUC of the SVM model was 0.691 (SD = 0.078), and the accuracy was 0.613 (SD = 0.074). The AUC of the Logistic regression model was 0.685 (SD = 0.093), and the accuracy was 0.667 (SD = 0.068). Comprehensive assessment shows that the XGBoost and LightGBM models outperform the traditional Logistic regression model in differentiating vulnerable carotid plaques, demonstrating a certain degree of model discrimination. Details are shown in Table 5. Clinical utility was evaluated by ROC analysis and decision curve analysis (DCA). ROC curves revealed that XGBoost and LightGBM attained higher AUCs than Logistic Regression in both the training and validation sets (Figure 7). The DCA curve shows that within a specific threshold probability range, the net benefits of the XGBoost and LightGBM models are superior to those of the traditional regression model, suggesting that the combination of tongue image parameters and blood metabolic markers has certain preliminary exploratory significance in differentiating coronary heart disease with vulnerable carotid plaques. However, its clinical net benefits still need to be confirmed by prospective studies, the net benefits of the XGBoost and LightGBM models show certain clinical applicability (Figure 8).

**Table 5:** Results on the classification of machine learning model on validation set.

Model	AUC (SD)	Accuracy (SD)	Sensitivity (SD)	Specificity (SD)	F1Score (SD)	Kappa (SD)
Logistic	0.685 (0.093)	0.667 (0.068)	0.488 (0.149)	0.760 (0.077)	0.494 (0.114)	0.248 (0.159)
XGBoost	0.717 (0.105)	0.738 (0.034)	0.256 (0.084)	0.988 (0.018)	0.394 (0.109)	0.294 (0.102)
LightGBM	0.727 (0.090)	0.751 (0.041)	0.323 (0.095)	0.973 (0.039)	0.463 (0.112)	0.346 (0.117)
SVM	0.691 (0.078)	0.613 (0.074)	0.659 (0.124)	0.587 (0.114)	0.536 (0.081)	0.224 (0.129)

### 3.6 Feature importance

**Figure 9:**
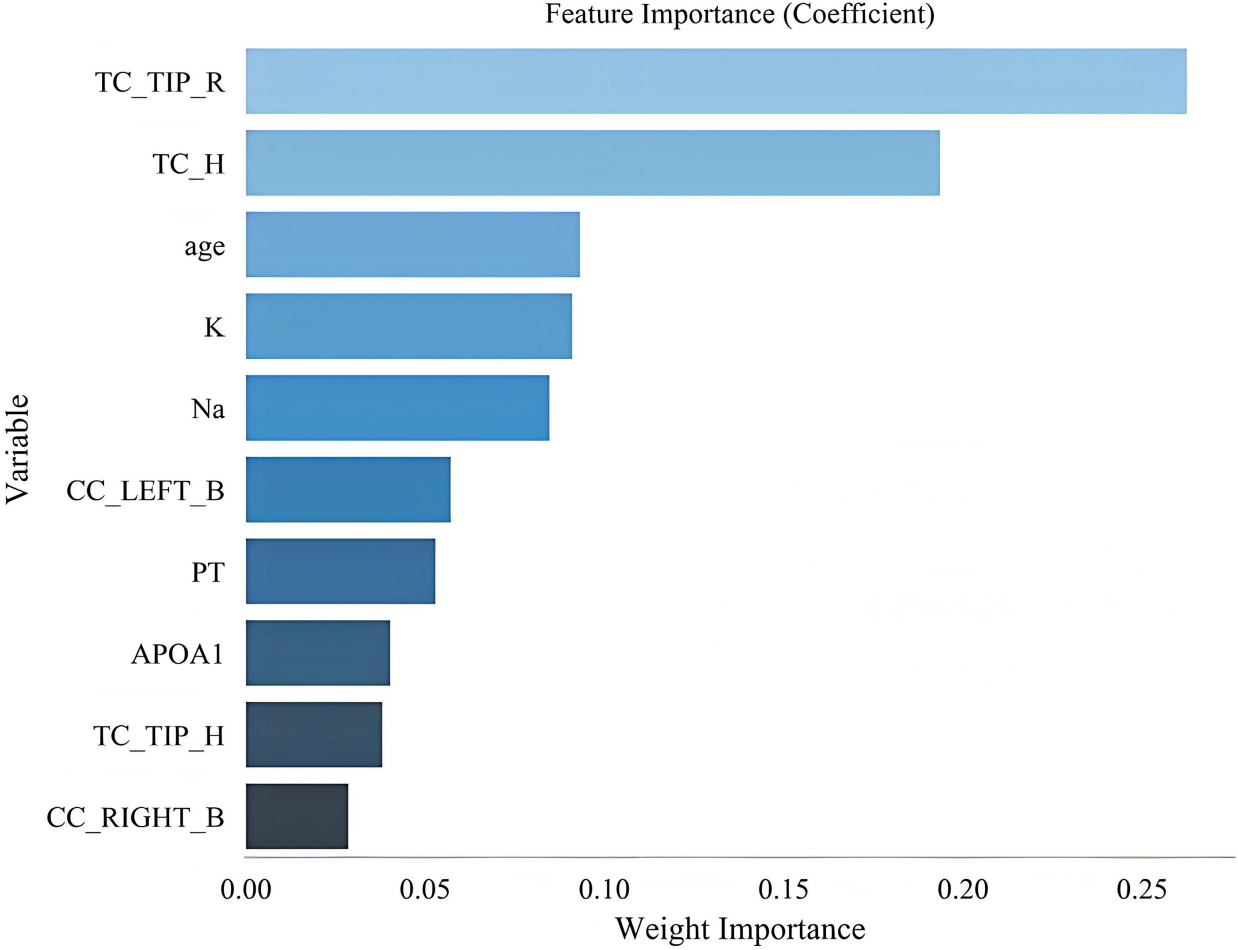
Feature importance ranking of the LightGBM model.

To assess the contribution of each feature variable to the model, this study ranked the feature importance of the best-performing LightGBM model. The results indicate that parameters derived from tongue images are significant to the model. Notably, the R value at the tip of the tongue and the overall H value of the tongue exhibit relatively high importance scores, implying that tongue color parameters possess diagnostic value in distinguishing plaque vulnerability. Additionally, blood metabolism indicators, including K, Na, PT, and APOA1, contribute to the model's performance. However, the combined use of tongue image parameters and blood metabolism markers provides limited incremental information regarding diagnostic performance, suggesting that this combined approach holds preliminary exploratory value. The contributions of the top 10 variables are shown in Figure 9.

### 3.7 Performance comparison of models with different feature combinations

**Figure 10:**
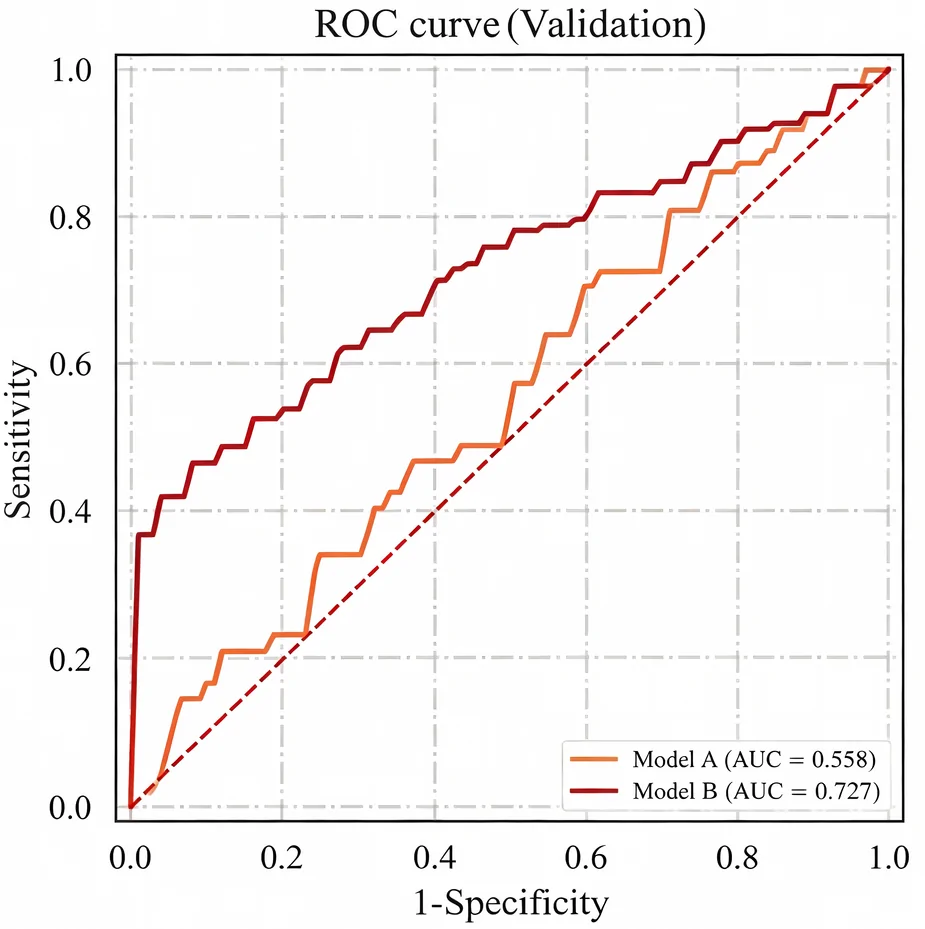
ROC curves of the baseline model and combined model.

To further clarify the incremental diagnostic value of tongue image parameters based on metabolic indicators, this study constructed two LightGBM models based on the feature variables screened out by LASSO regression for performance comparison in the validation set: Model A: Included age and blood metabolic indicators (APOA1, FBG, K^+^, Na^+^, PT, a total of 6 variables); Model B: On the basis of the basic model, tongue image parameters (TC_TIP_R, TC_TIP_H, TC_H, CC_MID_G, CC_TIP_B, CC_LEFT_B, CC_RIGHT_B, CC_B, a total of 14 variables) are further incorporated. The performance comparison of the validation sets of the two groups of models is shown in Table 6, and the ROC curves of the two groups of models are shown in Figures 10.

**Table 6:** Comparison of validation set performance between the baseline model and the combined model.

Model	AUC	Accuracy	Sensitivity	Specificity	F1Score
Model A	0.558	0.590	0.340	0.697	0.333
Model B	0.727	0.751	0.323	0.973	0.463

## 4 Discussion

This study preliminarily explored the application value of tongue image parameters combined with blood metabolic markers in the diagnosis of carotid vulnerable plaques in patients with coronary heart disease. By constructing multiple machine learning models and comparing their diagnostic efficacy, it provided a new non-invasive assessment method for cardiovascular risk stratification. The research results show that there is a correlation between tongue image parameters and multiple blood metabolism markers. The LightGBM model constructed based on the 14 characteristic variables screened out by LASSO regression demonstrates a certain discriminatory ability in the validation set, which is superior to the traditional Logistic regression model. The combined application of tongue image parameters and blood metabolic markers shows a numerical improvement in the diagnostic performance of the model, but its incremental value still needs further verification.

### 4.1 Clinical characteristics and pathophysiological basis of tongue manifestation parameters

This study found that compared with the non-vulnerable plaque group, the R, G, and B values in the middle of the tongue, tip of the tongue, right tongue, and the entire tongue region were significantly higher than those in the non-vulnerable plaque group (*P* < 0.01), suggesting a brighter tongue color. However, the S value and V value decreased significantly in multiple tongue body parts, indicating that the tongue color was brighter and the saturation was increased. Vulnerable plaques imply a higher inflammatory response and instability. From a traditional Chinese medicine perspective, this may be related to the pathological transformation of “long-term blood stasis turning into heat” or “toxins damaging the meridians”. The inflammatory response may manifest as a “hot image” on the tongue, causing the tongue color to appear relatively redder and brighter, which in turn leads to an increase in the RGB values. This reflects that when the disease worsens or turns to an unstable state, the tongue appearance also undergoes dynamic changes. Zhou's study on tongue image parameters of patients with carotid atherosclerosis found that the RGB values on the right side of the tongue in the plaque group were all increased (25), which is consistent with the findings of this study to some extent. However, this study is a cross-sectional study and cannot infer causal relationships. The above explanations are only preliminary speculations based on the existing knowledge framework. Analysis of tongue coating color parameters showed that the vulnerable plaque group had significantly higher R values across multiple tongue regions, and G and B values were also significantly elevated in most regions (*P* < 0.05), consistent with a redder coating in this group. This finding is consistent with previous research results (26, 27), further supporting the exploration value of tongue image parameters as potential phenotypic markers of the body's inflammatory and metabolic states. However, the inter-group comparison of tongue image color in this study was an exploratory analysis, and its results need to be verified in an independent cohort.

### 4.2 Relationship between blood metabolic biomarkers and plaque vulnerability

This study found significant group differences in APOA1, FBG, AST, K^+^, Na^+^, BUN, PT, and APTT (*P* < 0.05). The vulnerable plaque group exhibited higher FBG and AST, indicating greater metabolic disturbance in that cohort. LASSO regression identified APOA1, FBG, K^+^, Na^+^, and PT as primary variables associated with plaque vulnerability. APOA1 mediates reverse cholesterol transport by moving excess cholesterol from macrophages to the liver for clearance. Lower apolipoprotein A1 levels can accelerate macrophage foam cell formation within plaques, enlarge the lipid core, and increase vulnerability (28). Elevated FBG damages the vascular endothelium via mechanisms such as polyol pathway activation, protein kinase C activation, and advanced glycation end-product formation, thereby promoting plaque instability (29). Hypertension, a frequent comorbidity in CHD, exerts continuous stress on the vascular endothelium and thereby influences plaques and their fibrous caps (30). High sodium intake locally activates the angiotensin II system, which raises blood pressure and directly stimulates vascular smooth muscle cell proliferation and collagen remodeling, thereby weakening fibrous-cap mechanical stability (31). Prothrombin activation participates in fibrous-cap repair but also drives carotid plaques toward a vulnerable phenotype and elevates ischemic stroke risk via pro-inflammatory, pro-angiogenic, and pro-thrombotic pathways (32). Carotid atherosclerosis risk typically increases with age; however, in this study, CHD patients with vulnerable carotid plaques were younger, a finding that may reflect the relatively small sample size and the overall older age distribution of the cohort.

### 4.3 Comparison of machine learning performance

This study used four machine learning algorithms to build diagnostic models. XGBoost and LightGBM achieved perfect fit on the training set (AUC = 1.000) and retained relatively high diagnostic performance on the validation set (AUC = 0.717 and 0.727, respectively), markedly outperforming conventional Logistic Regression (AUC = 0.685). These results indicate that ensemble learning methods better handle high-dimensional data and capture nonlinear feature relationships (33). As representative gradient-boosting tree algorithms, XGBoost and LightGBM iteratively optimize loss functions and include regularization to reduce overfitting, and they handle missing values and outliers effectively, which suits the properties of medical data (34). Despite the strong model performance, overfitting was evident, likely because the relatively small sample size cannot support the models' complexity. In addition, tongue manifestation parameters are high-dimensional and noisy; although we applied LASSO for feature selection, image acquisition remains vulnerable to lighting, camera angle, and segmentation precision, and those measurement errors can be learned by the model and cause deep tree splits on noisy features that lead to overfitting. Although the SVM model achieved excellent performance in the training set (AUC = 0.891), its generalization in the validation set was slightly lower than that of XGBoost and LightGBM (AUC = 0.691); this difference may reflect SVM's sensitivity to kernel choice and the complexity of parameter optimization (35).

In this study, XGBoost and LightGBM achieved an AUC of 1.000 in the training set, but dropped to approximately 0.72 in the validation set, suggesting significant overfitting. This phenomenon is not uncommon in medical machine learning research, especially under the conditions of high-dimensional features (more than 130 features were initially included in this study), limited samples (*n* = 523), and data with measurement noise. When dealing with high-dimensional features, tree models tend to learn random noise as effective signals, resulting in inflated training performance. Although this study employed LASSO dimensionality reduction and ten-fold cross-validation, the stability of LASSO's feature screening on new data is still constrained by the sample size. It should be emphasized that overfitting does not completely deny the value of this study—the AUC of the LightGBM model in the validation set reached 0.727, and the feature importance ranking showed that the tongue image parameters (tip R value, overall tongue H value) ranked high, suggesting that there is a certain association between tongue image parameters and plaque vulnerability. Future research should be verified by using more rigorous regularization and simplification models in larger samples. In addition, the sensitivity of the LightGBM model in this study is relatively low, only 0.323. As a screening tool for vulnerable plaques, this means that more than two-thirds of high-risk patients will be missed, and it is completely unfeasible for clinical application under the current situation. The correlation signals between tongue image parameters and metabolic indicators deserve attention, but the current model is still far from being applicable for clinical decision-making.

This study further compared the incremental predictive value of tongue image parameters through feature grouping modeling. After adding tongue image parameters on the basis of age and metabolic indicators, the AUC of the validation set increased from 0.558 to 0.683, and the sensitivity increased from 0.340 to 0.362, indicating that tongue image parameters may provide additional predictive information on the basis of metabolic indicators. This finding is consistent with the result that there is only a weak correlation between tongue image parameters and metabolic indicators. The weak correlation implies a certain degree of information independence between the two, but due to the limited sample size, this independence is not yet sufficient to translate into statistically significant predictive gains. Despite this, tongue image parameters rank among the top in the feature importance ranking of the LightGBM model, and the combined model is numerically superior to the metabolic index model, suggesting that tongue image parameters still have certain exploration value as non-invasive and convenient auxiliary evaluation indicators. Future research needs to expand the sample size to further clarify its incremental effect.

### 4.4 Advantages and limitations

This study constructed four machine learning LASSO models and initially combined tongue image parameters with blood metabolic markers to predict vulnerable carotid plaques in coronary heart disease, achieving certain results. However, the following limitations still exist: Firstly, this study is a retrospective one, and there may be selection bias and information bias, making it impossible to completely rule out the influence of unmeasured confounding factors on the results. In addition, in this study, both ultrasound physicians received unified training and were evaluated in accordance with standard operating procedures. However, we did not systematically record the independent judgment results of each physician, so the Kappa value could not be calculated in this retrospective analysis. Secondly, the sample size of this study is limited, with only 204 cases in the vulnerable plaque group, which may have an impact on the stability and generalization ability of the model. Thirdly, without collecting patients' medication information, it is impossible to correct the impact of drugs on metabolic indicators. Fourth, although the tongue image collection process adopts a standardized procedure, factors such as lighting conditions and shooting angles may still have an impact on tongue image parameters. Fifth, It is important to emphasize that this study did not incorporate an external validation cohort, leaving the model's stability across diverse populations uncertain. Furthermore, the model's inadequate sensitivity hampers its ability to fulfill current clinical decision-making requirements. This investigation represents a preliminary exploration of predicting vulnerable plaques through the integration of tongue images and metabolites. Therefore, in future research, it is necessary to improve the medication record in a prospective multi-center design, expand the sample size, and conduct temporal validation or external validation to test the stability of the model and the net clinical benefit.

## 5 Conclusion

This study conducted a retrospective study to preliminarily analyze the differences between tongue image parameters and blood metabolic markers in patients with coronary heart disease accompanied by vulnerable carotid plaques, as well as the correlation between tongue image parameters and blood metabolic markers. The combined model of tongue image parameters and blood metabolic markers showed moderate predictive efficacy (AUC = 0.727) in the validation set, but its current sensitivity is relatively low and it does not yet meet the conditions for independent clinical application. There is a weak correlation between tongue image parameters and metabolic indicators, suggesting that the two may provide partially complementary information. This study provides preliminary exploratory evidence for the potential value of objective parameters in traditional Chinese medicine tongue diagnosis in the assessment of vulnerable carotid plaques in coronary heart disease. In the future, prospective large-sample studies need to be conducted to further verify the generalization ability of the model and explore the biological mechanisms behind tongue image changes.

## Data Availability

The datasets presented in this study can be found in online repositories. The names of the repository/repositories and accession number(s) can be found in the article/Supplementary Material.
