# Supplementary material for: Carotid vulnerable plaque in coronary heart disease: a machine learning-based diagnostic model integrating tongue parameters and blood metabolic biomarkers
Source: Front Cardiovasc Med. 2026 Sep 1;13:1852366. doi: 10.3389/fcvm.2026.1852366 (PMC13574680; doi:10.3389/fcvm.2026.1852366)
Supplement: Supplementary file 1 [file Datasheet1.docx]

**Supplementary Table 1. Summary of reproducibility parameters for machine learning modeling in this study**

| Item | Details |
| --- | --- |
| Sample size | Training set: 313 cases (vulnerable plaque group: 257, non-vulnerable plaque group: 56)  Test set: 210 cases (vulnerable plaque group: 169, non-vulnerable plaque group: 41) |
| Total number of variables | 104 (including clinical characteristics, tongue parameters, and blood metabolic indicators) |
| Missing data | See Supplementary Table S3 (summarizing the number and proportion of missing cases for each variable) |
| Missing data imputation method | Multiple Imputation by Chained Equations (MICE) with Predictive Mean Matching (PMM); continuous variables were imputed 5 times, and the mean value was taken as the final imputed value |
| Random seed | Fixed at 42 uniformly across all procedures (data splitting → imputation → modeling → cross-validation, consistent throughout) |
| Data splitting | Stratified random split into training set and validation set at a 6:4 ratio (maintaining proportional balance between groups); 10-fold stratified repeated cross-validation was adopted |
| Feature selection method | LASSO regression (Least Absolute Shrinkage and Selection Operator) with the λ.min criterion, and the optimal penalty coefficient was selected via 10-fold cross-validation |
| Model training framework | All models were built based on scikit-learn 1.2.2 + XGBoost 1.7.5 + LightGBM 3.3.5 |

Supplementary Table 2. Hyperparameter grid search for the four machine learning algorithms

| Hyperparameter | XGBoost | LightGBM | SVM | Logistic Regression |
| --- | --- | --- | --- | --- |
| n_estimators | - | 100 |  |  |
| max_depth | - | -1 |  |  |
| learning_rate | - | 0.1 |  |  |
| subsample | - | 1 |  |  |
| colsample_bytree | - | 1 |  |  |
| num_leaves |  | 31 |  |  |
| min_child_weight | - | 20 |  |  |
| min_data_in_leaf |  |  |  |  |
| C（regularization coefficient） |  |  | 1 | 1 |
| Gamma  （RBF kernel coefficient） |  |  | scale |  |
| kernel |  |  | rbf |  |
| penalty |  |  |  | l2 |
| C  （LR regularization coefficient） |  |  |  |  |
| solver |  |  |  | lbfgs |
| max_iter |  |  | -1 | 100 |

**Supplementary Table 3. Summary of missing data in the supplementary materials**

| Variable Category | Variable Name | Number of Missing Cases | Missing Rate |
| --- | --- | --- | --- |
| Blood indicator | TC | 26 | 4.9% |
| Blood indicator | TG | 38 | 7.2% |
| Blood indicator | HDL-C | 40 | 7.6% |
| Blood indicator | LDL-C | 39 | 7.4% |
| Blood indicator | APO-A1 | 117 | 22.3% |
| Blood indicator | FBG | 20 | 3.8% |
| Blood indicator | HbA1c | 63 | 12.0% |
| Blood indicator | K⁺ | 16 | 3.0% |
| Blood indicator | Na⁺ | 16 | 3.0% |
| Blood indicator | Cl- | 16 | 3.0% |
| Blood indicator | BUN | 12 | 2.2% |
| Blood indicator | Cr | 12 | 2.2% |
| Blood indicator | UA | 12 | 2.2% |
| Blood indicator | D-dimer | 31 | 5.9% |
| Blood indicator | PT | 20 | 3.8% |
| Blood indicator | APTT | 22 | 4.2% |
| Blood indicator | PTA | 344 | 65.7% |
| Blood indicator | INR | 20 | 3.8% |
| Blood indicator | FIB | 26 | 4.9% |

Supplementary Table 4. DeLong test results for baseline models and the combined model

| Model | AUC（95%CI） | *P*（DeLong test） |
| --- | --- | --- |
| Model A | 0.731(0.550-0.912) | NA |
| Model B | 0.684(0.493-0.873) | 0.419 |
